# Accumulation patterns of Cr in *Callitriche* organs—qualitative and quantitative analysis

**DOI:** 10.1007/s11356-015-5499-y

**Published:** 2015-10-06

**Authors:** Joanna Augustynowicz, Zbigniew Gajewski, Anna Kostecka-Gugała, Paweł Wróbel, Anna Kołton

**Affiliations:** Faculty of Biotechnology and Horticulture, Institute of Plant Biology and Biotechnology, Unit of Botany and Plant Physiology, University of Agriculture in Kraków, al. 29 Listopada 54, 31-425 Kraków, Poland; Faculty of Biotechnology and of Horticulture, Institute of Plant Biology and Biotechnology, Unit of Biochemistry, University of Agriculture in Kraków, al. 29 Listopada 54, 31-425 Kraków, Poland; Faculty of Physics and Applied Computer Science, AGH University of Science and Technology, al. Mickiewicza 30, 30-059 Kraków, Poland

**Keywords:** *Callitriche*, Chromium, EPR, Macrophytes, Phytoremediation, XRF

## Abstract

The aims of this study were both the qualitative and quantitative analysis of chromium accumulation in the shoots of *Callitriche cophocarpa*. This globally distributed, submersed macrophyte exhibits outstanding Cr phytoremediation capacity in an aquatic environment. Cr was applied separately for 7 days at two stable forms as Cr(VI) and Cr(III), known from their diverse physicochemical properties and toxicities. The maps of Cr depositions in young leaves, mature leaves, and stems were obtained by micro X-ray fluorescence spectroscopy (μXRF). The detailed analysis of XRF maps was done based on Image-Pro PLUS (Media Cybernetics) software. Cr was accumulated either in trichomes or vascular bundles in respect to the element speciation and the plant organ. The concentration of Cr significantly increased in the following order: Cr(VI) mature leaves < Cr(VI) young leaves = Cr(VI) stems < Cr(III) young leaves ≤ Cr(III) mature leaves ≤ Cr(III) stems. The observed differences in distribution and accumulation of Cr were correlated with the different reduction potential of Cr(VI) by particular plant organs. The reduction of Cr(VI) is considered the main detoxification mechanism of the highly toxic Cr(VI) form. The unique *L*-band electron resonance spectrometer (*L*-band EPR) was applied to follow the reduction of Cr(VI) to Cr(III) in the studied material.

## Introduction

Cr(III) and Cr(VI) are the two common and most stable forms of Cr in the environment. However, these two forms are principally different in their physicochemical properties and toxicities. Cr(VI) is found mainly in the form of chromate (HCrO_4_^−^) or dichromate (CrO_4_^2−^) anion in solutions. It is an extremely strong oxidant, highly soluble within a wide range of pH, and it is easily bioavailable. Its presence in cells results in the oxidation of unsaturated bonds in fatty acids, nucleic acids, and proteins. It shows mutagenic and cancerogenic effects on humans and animals (Kotaś and Stasicka 2000; Saha et al. 2011). It is also highly toxic to plants (Zayed and Terry 2003). Cr(III) is present in cationic forms in solutions (i.e., Cr(OH)_2_^+^). It is less toxic than Cr(VI) (Kotaś and Stasicka 2000). Trivalent Cr, in low concentrations, is a microelement in the diet of mammals, which is indispensable in removing surplus glucose (it is a component of the “glucose tolerance factor”) (Schwartz and Mertz 1959). However, when Cr(III) is present in higher concentrations, its harmful effects are associated with binding to functional groups of enzymes. It can also displace native ions in other biomolecules in a cell, resulting in changes in their structure and functions (Appenroth 2010; Codd et al. 2001; Kotaś and Stasicka 2000; Saha et al. 2011; Zayed and Terry 2003). Chromium is widely applied, e.g., in galvanization and tanning. Its salts possess attractive colors (violet, green, yellow, and orange), therefore, chromium is also used in producing various types of pigments (Kabata-Pendias and Muckherjee 2007). Chromium pollution constitutes a significant issue both in developed (e.g., the USA) and developing countries (e.g., India) (BlackSmith Institute 2015). In Poland, the highest levels of chromium contamination occur in the southern regions of the country, as a consequence of many tanneries operating there for centuries (Ślusarczyk et al. 2010). Both Cr(VI) and Cr(III) compounds are qualified by the US Environmental Protection Agency (US EPA) as priority toxic pollutants.

Why did we use *C. cophocarpa* for this study? This aquatic higher plant is an efficient Cr phytoremediator in water, as we proved in earlier studies (Augustynowicz et al. 2010, 2013b). Phytoremediation is an alternative to the physicochemical methods in cleaning up the environment. It denotes the use of plants to remove pollutants from water, soil, and air and/or transform these pollutants into less harmful forms (Ali et al. 2013). In accordance with the definition of hyperaccumulation (Van der Ent et al. 2013), when it grows in the natural environment, *C. cophocarpa* would be called a Cr hyperaccumulator. *C. cophocarpa* is a perennial macrophyte, the most common representative of the *Callitriche* genus in Poland and elsewhere in Europe (Schotsman 1972; Zając and Zając 2001). It grows both in stagnant and running water. Of prime importance for phytoremediation studies is that *C. cophocarpa* is a submerged plant. Submerged aquatic plants possess a surface of contact with the polluted environment that is markedly larger than emergent or free-floating plants. Moreover, the ability of *Callitriche* to accumulate Cr in shoots is also of direct significance to the environment. It is associated with the higher probability of removing undamaged shoots than undamaged roots.

In our earlier work (Augustynowicz et al. 2014), we demonstrated the different accumulation patterns of Cr in *Callitriche* mature leaves and stems incubated both in Cr(III) and Cr(VI) solutions. In the present paper, we focus on the differences between the young and mature organs. Firstly, we performed both a quantitative and qualitative analysis of Cr accumulation using young leaves and mature organs (mature leaves and stems). For that reason, we used micro X-ray fluorescence spectroscopy (μXRF), which is a non-destructive technique that allows the pattern of elemental distribution to be determined in micrometer scale by the use of a very narrow beam of X-rays. With this technique, it is possible to perform a qualitative and quantitative analysis at concentration levels in the μg · g^−1^ range (Punshon et al. 2009) at the same time. The intensity of the recorded fluorescence signal of Cr was proportional to the element content. With respect to the earlier work, a diverse computation method was used in the present study. The main novelty of this work, however, was the use of electron paramagnetic resonance spectroscopy (EPR) to track the reduction of Cr(VI) in the studied samples. *L*-band EPR permitted us to measure the degree of Cr(VI) reduction via the recording signal coming from Cr(V), an unstable radical being an intermediate product in the redox reaction: Cr(VI) ↔ Cr(III). Reduction of Cr(VI) to Cr(III) is regarded as a principal mechanism of Cr(VI) detoxification. It results from the markedly lower toxicity of Cr in the trivalent form (Kotaś and Stasicka 2000). The application of 1.2 GHz frequency (*L*-band) in EPR spectroscopy gives the ability to measure the signal of paramegnetic Cr(V), which is an unstable intermediate upon reduction of Cr(VI) to stable Cr(III). In such conditions, Cr(III) is not detectable. The *L*-band EPR technique, in contrast to the most common used *X*-band EPR, enables hydrated sample measurement and in vivo (real time) studies. We found that the reduction capacity of Cr(VI) is responsible for the different accumulation quantity and different Cr accumulation patterns in studied organs.

This work has contributed to knowledge concerning the mechanisms of the response of aquatic phytoremediators to elevated concentrations of Cr ions. It also provides information concerning the application of particular organs of *C. cophocarpa* in efficient Cr remediation.

## Material and methods

### Plant material and incubation in Cr solutions

*C. cophocarpa* was collected from natural stands located in an area of southern Poland (N 50° 15′ 58″ / E 19° 56′ 24.9″; N 50° 14′ 32.9″ / E 20° 03′ 55″) during the vegetation seasons of 2012 and 2014. Ten-to-fifteen-centimeter-long shoots were rinsed several times with tap water and then rinsed a few times in distilled water. The plants were then incubated for 7 days in Cr solutions based on filtered (Milipore filters, 0.2 μm pore size) river water derived from the plant’s natural stand. The solutions contained 100 μM (5.2 mg dm^−3^) of Cr(VI) or Cr(III) and were prepared from K_2_Cr_2_O_7_ or Cr_2_(SO_4_)_3_ · 18H_2_O (POCh Gliwice, Poland) for Cr(VI) or Cr(III), respectively. The concentration of Cr ions was chosen according to the earlier experiments (Augustynowicz et al. 2010). This amount of Cr caused only minor physiological stress to the plants with no significant disorders. Inductively coupled plasma mass spectrometer (ICP-MSELAN 6100, Perkin Elmer) (PN-EN ISO 9963–1:^2001^) and titration methods (PN-ISO 9297:^1994^, PN-EN ISO 17294–1:^2007^) were applied to analyze the chemical composition of water. The spectrometer was calibrated with the ICP multi-element standard (Merck). The concentrations of ions (mg dm^−3^) present in the water were the following: 4.24 Na^+^, 1.75 K^+^, 69.65 Ca^2+^, 5.01 Mg^2+^, 2 · 10^−3^ Fe^2+^, 5 · 10^−3^ Mn^2+^, 5 · 10^−3^ Zn^2+^, 6 · 10^−4^ Cu^2+^, 10^−3^ Mo^6+^,16.50 Cl^−^, 10.20 SO_4_^2−^, 189.00 HCO_3_^2−^, 13.50 NO_3_^2−^, 0.15 PO_4_^3−^, and 0.08 BO_3_^3−^. The level of Pb, Hg, Cd, Tl, and Ni did not exceed 0.1 μg dm^−3^. Cr content was lower than 0.02 μg dm^−3^. The average electrical conductivity of water was equal to 0.3 mS cm^−1^, pH to 7.8 and Eh (redox potential) to 180 mV. A weight of 1.5 g of shoots were cultured in 300 ml of the aforementioned Cr media or in the control solution (without Cr salts). The plant material was incubated in the phytotron under 16 h of light intensity at 35 μmol m^−2^ s^−1^ (LF 36W/54, Piła, Poland) and 8 h of darkness, at 23 °C. The light intensity was comparable to the one detected in *Callitriche*’s natural environment. The analysis of chromium distribution was performed on young leaves, mature leaves, and stems.

### Micro X-ray fluorescence spectroscopy

#### XRF measurements

**Fig. 1:**
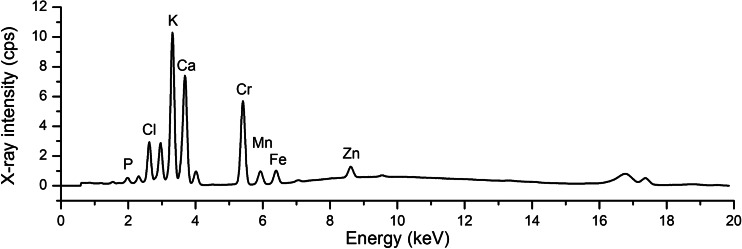
An average spectrum acquired for Cr(III)-treated mature leaf

After treatment with Cr(VI)- and Cr(III)-containing media the plant samples were prepared according to a freeze-drying protocol to avoid the dehydration and redistribution of Cr ions during prolonged μXRF measurements (Augustynowicz et al. 2014). The spatial distribution of chromium was investigated with a laboratory micro-XRF spectrometer (Wróbel et al. 2012). The spectrometer consisted of a low power 50 W X-ray tube with molybdenium anode integrated with a polycapillary lens (XOS, USA) and a silicon drift detector with a 10-mm^2^ active area (KETEK, Germany). The primary radiation from the X-ray tube was formed into a Gaussian-shaped beam with the size of 16.4 μm (measured as the full width at half maximum). The angle between the impinging beam and the sample surface was 45°, and the angle between the detector axis and the impinging beam was 90°. The plant specimens were mounted between two Mylar^®^ foils (with a thickness of 2.5 μm) stretched on a plastic ring. The investigation of elemental distribution was done by moving the sample surface across the primary X-ray beam with the use of a motorized stage (PI, Germany). The typical size of the investigated area varied from 1 to 1.5 mm^2^, whereas the step of movement in both horizontal and vertical directions was 20 μm. The spectrum acquisition time for each pixel varied between 1 and 1.5 s. The average time of imaging of the analyzed area was 4.5 h. An average spectrum with description of X-ray peaks of representative sample is presented in Fig. 1. As a result of the analysis of the spectra, the XRF maps were obtained showing the intensity of the characteristic X-ray emission of chromium (measured as counts per second (cps)) from the irradiated area (380 μm^2^). The intensity of X-ray emission was proportional to the element content. Thus, the intensity of the X-ray at the given energy typical for chromium, recorded by the SDD detector from the irradiated area (cps), was used to determine the content of the element in the studied organs of *C. cophocarpa.* Eight or three independent replicates of Cr-treated samples or control, respectively, were analyzed in three independent series of experiments.

#### Analysis of XRF maps

**Fig. 2:**
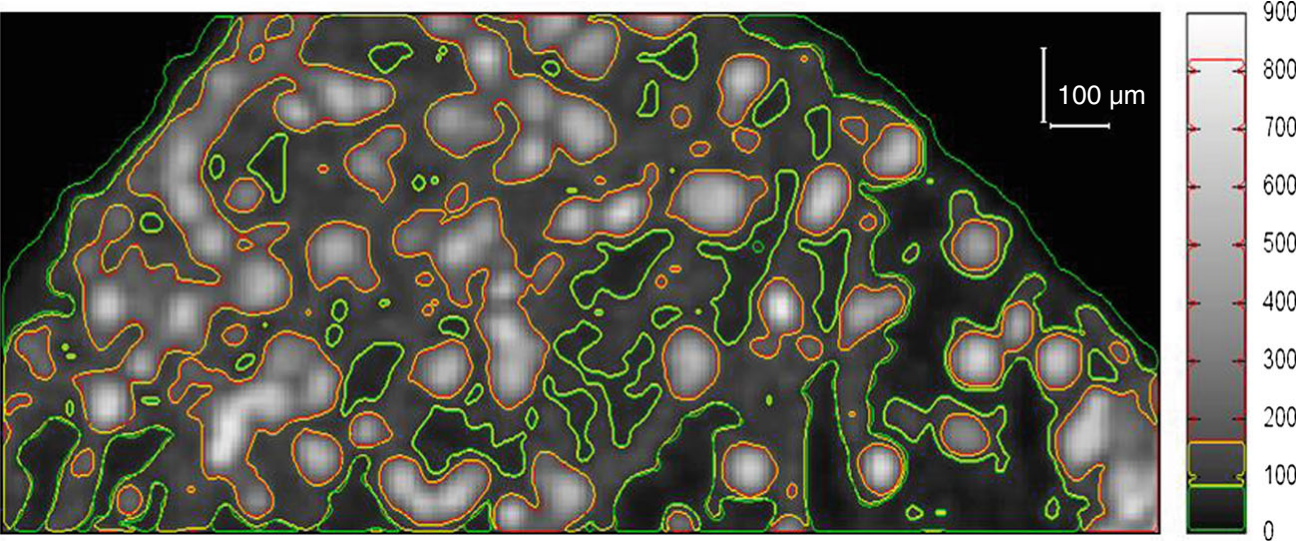
An example of the XRF map of chromium distribution in the *C. cophocarpa* mature leaf incubated in Cr(III) used for analysis on Image-Pro Plus software. *Different colors* point out areas of different Cr accumulation. The *black* and *white* scale on the right indicates X-ray intensity for Cr in counts per second (cps)

Two-dimensional maps of chromium distribution from the X-ray fluorescence spectroscopy were visualized as jpg/gif pictures. The analysis of XRF maps was conducted using Image-Pro Plus 4.0 (Media Cybernetics, Inc., USA) software. The intensity of grayness of each spot reflected the intensity of fluorescence for Cr. Before the analysis, each map was calibrated separately based on the individual linear length scale (number of pixels per length unit was set). The boundary value of the signal was estimated separately for each map, which was dependent on the scale resolution (maximum signal recorded in it) (Fig. 2).

### Microscopy

For the visualization of the leaf surface, scanning electron microscopy (HITACHI S-4700) was used. Prior to microscopy observation, the leaves were fixed in 5 % glutaraldehyde in a phosphate buffer (pH 7.2). The samples were then dehydrated in serial dilutions of ethanol and acetone, after which they were critical-point dried in liquid CO_2_ and coated with gold using a JEOL-JFC 1100E (Japan) sputter coater. The specimen was kindly prepared and visualized by Dr. Bartosz J. Płachno (Department of Plant Cytology and Embryology, Jagiellonian University, Kraków, Poland).

### Electron paramagnetic resonance spectroscopy

The reduction of Cr(VI) by the studied plants was measured in vivo using a custom-built electron paramagnetic resonance (EPR) spectrometer (*L*-band, 1.2 GHz). This apparatus can detect a signal of the highly unstable Cr(V) intermediate that is generated upon Cr(VI) → Cr(III) reduction. Prior to the experiments, the plants were washed with distilled water twice and drained gently with filter paper. The sample for EPR measurements was prepared by immersing 0.4 g of the material into 1.5 ml of a dichromate solution. The solution was based on twice-diluted macro- and microelements of a standard MS medium, pH 5.4, supplemented with 1 mM (5.2 mg dm^−3^) Cr(VI) (as K_2_CrO_4_; POCh Gliwice, Poland). The composition of the medium was chosen based on preliminary tests (data not shown). The signal was recorded immediately after preparing the sample. The EPR analyses were carried out with typical settings: maximum microwave power of 16 mW, 33.8 kHz field modulation frequency, sweep range 35 G, sweep time 20 s, and a time constant of 10 ms. The presented data were analyzed using custom-designed computer software. Two independent sets of experiments were performed. In each set, three independent samples were prepared and measured as individual runs. Each run was obtained by the averaging of 20 individual scans.

### Statistics

The results were analyzed using one-way ANOVA/Student’s *t* tests to compare the differences between the samples based on the STATISTICA ver. 10 software (StatSoft Inc. 2011). Following the rejection of the null hypothesis, LSD-Fisher’s or Tukey’s tests were performed to determine the statistical significance of the results (*α* = 0.05).

## Results and discussion

**Fig. 3:**
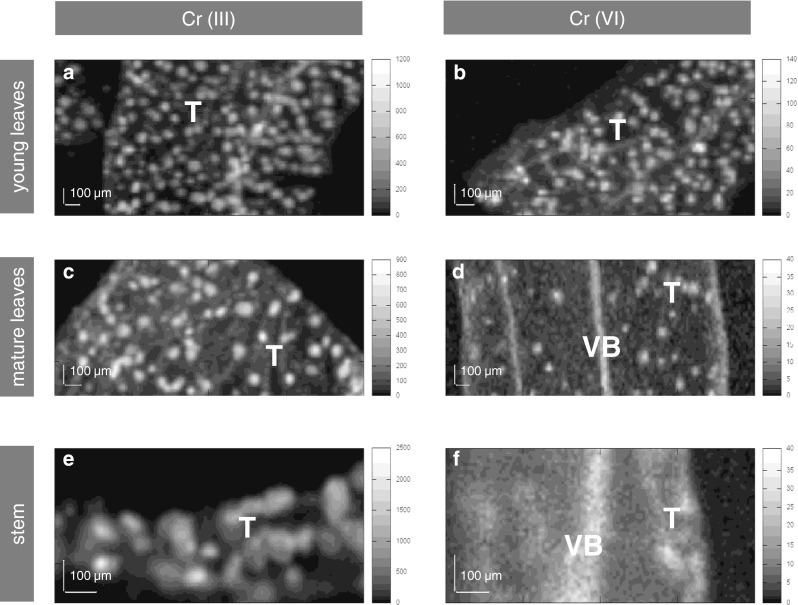
Representative XRF maps of Cr accumulation patterns in studied organs. **a** Cr(III)-treated young leaves; **c** Cr(III)-treated mature leaves; **e** Cr(III)-treated stems; **b** Cr(VI)-treated young leaves; **d** Cr(VI)-treated mature leaves; **f** Cr(VI)-treated stems. *Scale on the right* indicates X-ray fluorescence intensity for chromium in counts per second (cps). *VB* vascular bundle, *T* trichome

**Fig. 4:**
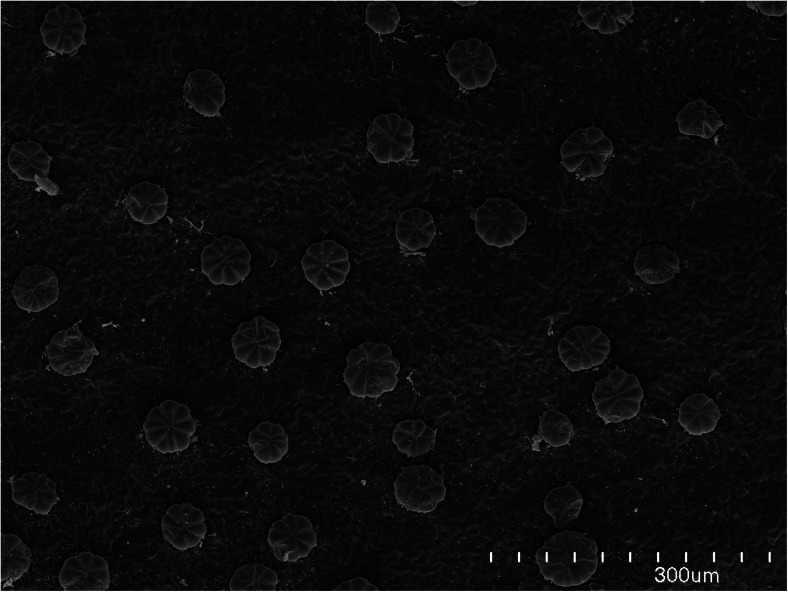
Trichomes on the leaf surface of *C. cophocarpa*. This specimen was kindly prepared and photographed by Dr. Bartosz J. Płachno, Jagiellonian University, Kraków, Poland

Distinct patterns of Cr arrangements were found with respect to Cr speciation as well as the plant organ (Fig. 3). Cr was found solely in spot-like structures in all investigated samples, i.e., young leaves (Fig. 3a), mature leaves (Fig. 3c), and stems (Fig. 3e), when the shoots were exposed to trivalent chromium. These spot-like structures corresponded to the trichomes located on the epidermis of the investigated specimen (Fig. 4), which was proved in our earlier work (Augustynowicz et al. 2014). Plants treated by hexavalent Cr exhibited diverse Cr localization except from young leaves, where this element was also found exclusively in trichomes (Fig. 3b). It must be stressed that young leaves showed identical Cr localization (in trichomes) regardless of the chromium speciation in the solution. Cr accumulation patterns were similar in mature leaves (Fig. 3d) and stems (Fig. 3f) treated by Cr(VI). In these organs, Cr was observed preferentially in vascular bundles but also in trichomes. Trichomes may play a secretion function (Lavid et al. 2001; Wagner 1991). However, we postulate that *C. cophocrapa*, when submersed in trivalent Cr solution, also used trichomes to adsorb chromium from the surrounding medium. This phenomenon was described for water lily accumulating cations of some heavy metals in their epidermal glands (Lavid et al. 2001).

**Fig. 5:**
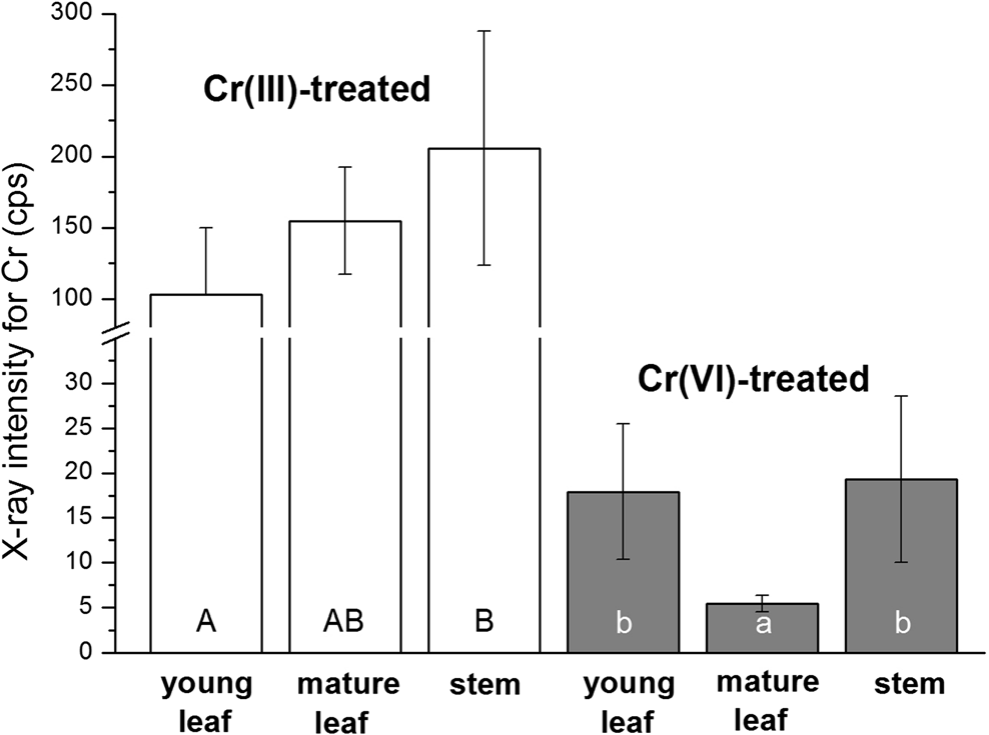
The mean values of X-ray intensities for Cr (cps) related to the element content in the studied samples. Different letters indicate statistically significant differences for Cr(III) and Cr(VI) separately according to ANOVA and Tukey’s tests. *Error bars* represent SDs; *n* ∈ <6; 8>

The statistically significant differences in the Cr fluorescence signals that are dependent on the Cr valency (*p* < 0.00001) and the type of plant organ (*p* < 0.020 for Cr(III) and *p* < 0.002 for Cr(VI)) were found. The average fluorescence signal reflected in the amount of Cr in the studied samples is presented in Fig. 5. Generally, the average fluorescence intensity for Cr detected in Cr(III)-treated samples was a magnitude higher than the one in the Cr(VI)-treated specimens. The average signal in control samples was negligible (1.3 ± 0.2 cps). Stems showed the highest (mean 205.5 cps) whereas young leaves showed the lowest (mean 103.2 cps) X-ray fluorescence signal for chromium in the group of Cr(III)-treated samples. In contrast to these results, a comparable intensity of X-ray fluorescence was found in the case of young leaves (mean 17.9 cps) and stems (mean 19.9 cps) influenced by Cr(VI). Mature leaves exposed to hexavalent Cr exhibited the lowest fluorescence (mean 5.4 cps) of all the samples tested. In summary, according to statistical significance analysis, the concentration of Cr expressed as Cr fluorescence intensity (cps) can be ordered as follows: Cr(VI) mature leaves < Cr(VI) young leaves = Cr(VI) stems < Cr(III) young leaves ≤ Cr(III) mature leaves ≤ Cr(III) stems. The high resolution of the μXRF method enabled us to measure the Cr fluorescence signal in single leaves. The analysis of the Cr concentration in single, highly hydrated leaves like *Callitriche* would be impossible using commonly applied techniques of atomic absorption spectrometry (like atomic absorption spectrometry (AAS) or inductively coupled plasma (ICP) membrane absorption spectrometry). The mentioned methods required a relatively high amount of sample biomass.

Trivalent chromium could be accumulated to a higher extent in aquatic plants than Cr(VI) (Zhang et al. 2007) that was also confirmed in our work. It is related e.g., to the easy binding of Cr^3+^ to functional groups like hydroxyl, carbonyl, carboxyl, amide, sulfhydryl and sulfonate from the plasmalemma and cell wall (Kotaś and Stasicka 2000; Mohan and Pittman 2006). The cell wall of mature organs like leaves and stems is thicker and has a different chemical structure when compared to young organs. It has more metal-binding sites, and thus a greater sorption capacity.

As presented in Fig. 3, young leaves of the two types: Cr(III)- and Cr(VI)-treated plants, exhibited the same Cr distribution patterns. However, the accumulation capacity of Cr differed in these two samples (compare Fig. 5). Furthermore, young leaves treated by hexavalent chromium showed Cr depositions only in trichomes, whereas the mature leaves and stems also deposited Cr in vascular bundles in this case. To elucidate the observed phenomenon, firstly, we measured the number of spots that reflects the number of hairs on the surface of mature and young leaves. The measurements were carried out on the flat surfaces of leaves because the geometry of stems (round in shape) made the measurements imprecise. We did not find statistically significant differences between the number of spot-like structures on the surface of young and mature leaves (*p* < 0.430; *n* = 6). The mean value of the total number of spot-like structures that accumulate Cr on the young leaves was 140 μm^−2^ (±14), whereas in the case of mature leaves, the value was 151 μm^−2^ (±32). Therefore, it can be concluded that the number of spot-like structures that accumulate Cr cannot be the reason for the observed different Cr accumulation patterns, i.e., in trichomes or preferentially in vascular bundles, in young and mature leaves treated by Cr(VI), respectively. There is no available information about the mechanisms controlling the distributions of trichomes on *Callitriche* leaves. Generally, the development of stomata, trichomes, or glands (secreting trichomes) and the determination of their distributions on the leaf epidermis have only been reported for a few model plant species (Larkin et al. 1996; Marks 1994; Sachs and Novoplansky 1993). Trichomes are formed very early in leaf development. Cell divisions occurring after the formation of the last trichomes can contribute to the distance between the adjacent trichomes on the mature leaf, but the number of trichomes does not vary between young and mature leaves. Lavid and coworkers (2001) showed, however, that the accumulation of metallic elements in the epidermal glands of water lilies depends on the structure of so-called cap cells. Mature leaves of water lilies with larger numbers of degenerated cap cells accumulated metallic elements to a higher extent than young leaves (Lavid et al. 2001). Further studies are needed to elucidate the exact mechanism of Cr accumulation in trichomes in ontogenetically different specimens of the *Callitriche* epidermis, which was not a subject of the present work.

**Fig. 6:**
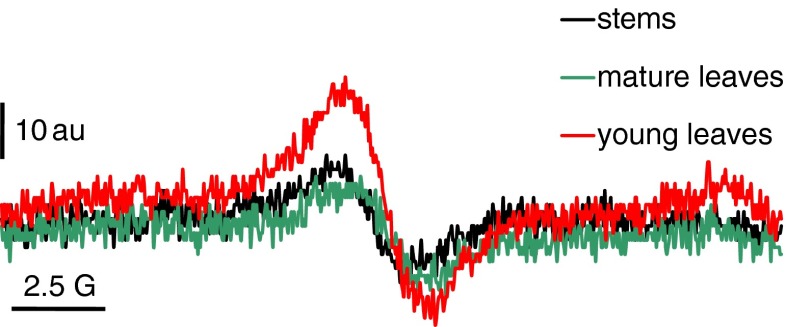
EPR signal of Cr(V) as a result of Cr(VI) reduction by the studied samples. *au* arbitrary unit, *G* Gauss

In the next step of our study we analyzed the Cr(VI) reduction potential by the particular specimen (Fig. 6). We wondered whether the different reduction capacity of the particular organs could be a reason for the diverse Cr accumulation capacity and patterns in different organs. To measure the reduction of Cr(VI), we applied *L*-band electron paramagnetic resonance spectroscopy (*L*-band EPR). Measurements could be made in an aquatic environment using a field frequency of 1.2 GHz, avoiding complicated preparation procedures. This technique, therefore, is excellent for analyzing in vivo reactions of paramagnetic substances that take place in tissues as well as in solutions or cell suspensions (Appenroth et al. 2000). EPR is one of the methods for studying chemical compounds possessing unpaired electrons (Płonka and Elas 2002). Thus, the *L*-band EPR permitted us to measure the degree of reduction of Cr(VI) via a recording signal coming from Cr(V), an unstable radical being an intermediate product in the reduction reaction: Cr(VI) → Cr(III). The degree of reduction of chromates correlated with the amplitude for the Cr(V) signal. The specimens were incubated in Cr(VI) solution, and the signal of Cr(V) accompanying Cr(VI) reduction was recorded. We found statistically significant differences (*p* < 0.00002) in the amplitudes of Cr(V) signals between samples (Table 1, Fig. 6). Hexavalent chromium was reduced in young leaves the most effectively; the mean amplitude of the Cr(V) signal was *ca.* 2-times higher than the one recorded for both mature leaves and stems (Table 1). The control plant samples, i.e., immersed in the solution free of chromium, showed no paramagnetic activity during the whole experiment (data not shown). The difference between the young and mature organs would be even higher if the dry weight of particular organs were considered; it is widely known that young organs are more hydrated than mature ones.

**Table 1:** The mean values of amplitudes of Cr(V) signal (au)

Specimen	Amplitude of Cr(V) signal (au)
Young leaf	31.8 ± 6.4 (a)
Mature leaf	16.0 ± 2.7 (b)
Stem	17.3 ± 2.8 (b)

Cr(V) signal resulted from Cr(VI) reduction by the studied samples. Different letters indicate statistically significant differences according to ANOVA and LSD-Fisher’s tests; *n* = 6

Direction of the redox reaction Cr(VI)↔Cr(III) depends on the presence of electron donors/acceptors and pH. There are only a few oxidants, like MnO_2_, H_2_O_2_, O_3_, and PbO_2_, which are able to mediate the oxidation of Cr(III) to Cr(VI) in the natural environment. On the other hand, reduction of Cr(VI) can occur under a variety of conditions in the environment, and the most important reducing agents are as follows: organic substances, SH_2_, S, FeS, NH_4_^+^ and NO_3_^−^ (Kimbrough et al. 1999; Kotaś and Stasicka 2000). In the organisms, the bio-reduction of Cr(VI) is carried out directly or indirectly outside or/and inside the cells. Reduction may be proceeded spontaneously with low-molecular weight compounds like: glutathione, cysteine, ascorbate, H_2_O_2_, NAD(P)H, and monosaccharides (Saha et al. 2011). Additionally, the redox reaction can be also catalyzed by soluble or membrane-associated enzymes in the case of microorganisms (Kanmani et al. 2012).

We can assume that as a result of the redox reactions taking place in *Callitriche* tissues, trivalent forms of chromium appear. The potential for Cr(VI) reduction in young leaves, however, is significantly higher than the potential found in mature organs. However, based on the results of our present work, we cannot explain the detailed mechanism of this phenomenon yet. The exhaustive biochemical analysis of the chemical nature of reducing agents produced by *Callitriche* shoots under Cr(VI) exposure are the subject of our current investigations. The redox reaction Cr(VI) → Cr(III) is probably carried out inside the plant tissue. The results obtained in our previous work (Augustynowicz et al. 2013a) indicated that the conversion of Cr(VI) occurs exclusively in the plant shoots. *Callitriche* did not secrete any reducing agents outside into a surrounding medium. On the other hand, no oxidation of Cr(III) to Cr(VI) was found in the *Callitriche* tissue. Based on the results obtained in the present study, we can hypothesize that Cr(VI) might be taken up by the plant into vascular bundles (compare Fig. 2d, f). Since we did not find high redox activity in stems and mature leaves, Cr(VI) can remain dissolved in the vascular bundles of these organs. Though, when reaching young leaves, Cr(VI) penetrates into trichomes, where it is actively reduced to Cr(III). Therefore, the patterns of Cr depositions in young leaves treated both by Cr(III) and Cr(VI) and mature leaves submersed in Cr(III) are indistinguishable. In contrast to young leaves, similar redox potential for Cr(VI) was found in mature leaves and stems; therefore, the accumulation pattern of Cr is similar in these organs. Reduction of Cr(VI) was detected only to some extent when they were exposed to Cr(VI). That is why Cr was visible both in the vascular bundles and some trichomes in these cases.

The young leaves of plants incubated in dichromate showed a relatively high Cr content in the group of Cr(VI)-treated specimens. On the other hand, when incubated in Cr(III), they showed a relatively low accumulation capacity for chromium when compared to mature organs. These results could be explained as follows. As has already been shown, young leaves actively reduced Cr(VI). At the same time, however, the physicochemical structure of their cell walls differs from the cell wall structure of mature organs. The phenomenon of local variation in wall thickness and composition is an integral part of cell growth and differentiation (Roberts 2001). When incubated in Cr(VI), young leaves actively reduced chromate to a trivalent form, which was finally accumulated to a higher extent.

## Conclusion

Different Cr accumulation patterns were visualized in *C. cophocarpa* shoots with respect to the particular plant organ and the chromium oxidation state. Young leaves treated by Cr(VI) exhibit identical Cr distribution to Cr(III)-influenced organs. We recommend using actively growing young shoots for the application of Cr(VI) detoxification in aquatic systems. Young leaves have both a high accumulation capacity and redox potential for Cr(VI). On the other hand, we advise utilizing mature plants with limited growth when Cr(III) is the dominating Cr species in polluted water. Cr(III) is accumulated to a higher extent by mature organs. Further analyses are needed to determine the highest activity of chromate reduction by this perennial macrophyte during growing seasons.
